# Interactome between ASFV and host immune pathway proteins

**DOI:** 10.1128/msystems.00471-23

**Published:** 2023-11-15

**Authors:** Qijun Wu, Yingying Lei, Ya Zuo, Ji Zhang, Fenglin Guo, Weize Xu, Tanghui Xie, Dang Wang, Guiqing Peng, Xiangru Wang, Huanchun Chen, Zhenfang Fu, Gang Cao, Jinxia Dai

**Affiliations:** 1 State Key Laboratory of Agricultural Microbiology, Huazhong Agricultural University, Wuhan, China; 2 College of Veterinary Medicine, Huazhong Agricultural University, Wuhan, China; 3 Hubei Key Laboratory of Agricultural Bioinformatics, College of Informatics, Huazhong Agricultural University, Wuhan, China; 4 Departments of Pathology, College of Veterinary Medicine, University of Georgia, Athens, USA; 5 Hubei Hongshan Laboratory, Wuhan, China; 6 College of Biomedicine and Health, Huazhong Agricultural University, Wuhan, China; Princeton University, Princeton, New Jersey, USA

**Keywords:** African swine fever virus, host immune pathway, protein-protein interaction, high-throughput yeast two-hybrid screening

## Abstract

African swine fever virus (ASFV) causes severe acute hemorrhagic disease with high mortality in domestic pigs but minimal or no symptoms to warthogs, for which the underlying mechanism remains elusive. ASFV encodes numerous proteins to disturb host immune responses via interacting with host proteins. In this study, we deciphered the comprehensive protein-protein interaction (PPI) network between ASFV and host immune pathways by the recombination-based library vs library high-throughput yeast two-hybrid screening. This PPI network contains both ASFV-host PPI and ASFV-ASFV PPI information, providing a comprehensive ASFV-host interactome landscape. We further explored the ASFV-host PPI difference between domestic pigs and warthogs. Moreover, the inhibitory effect of ASFV proteins in the PPI with cGAS-STING pathway on IFN-I and NF-κB promoter activity was screened to investigate the ASFV-host PPI functions. Our work will help the exploration of ASFV pathogenesis and the development of anti-ASFV vaccine and ASFV-resistant transgenic pigs.

**IMPORTANCE:**

African swine fever (ASF), caused by African swine fever virus (ASFV), has become a major crisis for the pork industry in recent years. The mechanism for ASFV pathology and the clinical symptoms difference of ASF between domestic pigs and reservoir hosts remain to be elucidated. We deciphered the comprehensive protein-protein interaction (PPI) network between ASFV and host immune pathways. The intensive PPI network contained both ASFV-host immune pathway PPI and ASFV-ASFV PPI information, providing a comprehensive ASFV-host interaction landscape. Furthermore, the ASFV-host PPI difference between domestic pigs and warthogs was explored, which will be instructive for exploring essential candidates involved in ASFV pathology. Moreover, we screened the inhibitory effect of ASFV proteins in the PPI with cGAS-STING pathway on IFN-I and NF-κB, further providing possible functions of ASFV-host PPI network in innate immune regulation.

## INTRODUCTION

African swine fever (ASF), caused by the African swine fever virus (ASFV), is a severe acute hemorrhagic disease with high fever, high mortality, and lymphoid tissue necrosis in domestic pigs (1, 2). To date, no safe and efficacious vaccine or antiviral drug is available for ASF disease (3). Intriguingly, bushpigs and warthogs infected by ASFV show minimal or no clinical signs but providing a reservoir of infection (4, 5), of which the underlying mechanism remains elusive. Elucidating the mechanism attributed to this difference will facilitate to understanding the pathogenesis of ASF and the development of anti-ASFV drug and vaccine. As a large, enveloped double-stranded DNA virus belonging to the family *Asfarviridae*, ASFV encodes more than 150 genes (6, 7). Besides the structural proteins for virus assembly, many ASFV proteins are involved in the evasion of host defense, DNA replication repair, as well as gene expression regulation (8). However, the function of the majority of ASFV genes has not been characterized, hampering the understanding on ASFV pathology, immune escaping mechanism, and vaccine/antiviral drug development.

To ensure efficient infection, ASFV has evolved numerous genes to disturb various host immune responses (9). To escape from innate immunity, ASFV encodes multiple proteins primarily inhibiting the actions of TLR3 and the cGAS-STING pathway to suppress *NF-κB* and *IFN-I* expression. It has been shown that ASFV A276R inhibits the recognition of TLR3 (10). ASFV I329L interacts with host TRIF and subsequently impedes the TLR3-mediated activation of NF-κB and the induction of IFN-β (10, 11). Meanwhile, multiple ASFV genes (*A137R, A528R, DP96R, A276R, MGF360-11L MGF360-12L, MGF360-14L, E120R, F317L, MGF505-3R, MGF505-7R, MGF505-11R, A104R,* and *I215L*) can suppress the cGAS-STING pathway and negatively regulate *IFN-I* expression by targeting IRF3, IRF7, IRF9, TBK1, IκB kinase beta (IKKβ), STAT1, STAT2, RNF138, or STING (10, 12–20). Intriguingly, CD2v (EP402R) can activate NF-κB and induce IFN signaling as well as apoptosis in swine lymphocytes/macrophages by interacting with CD58 (21). Furthermore, ASFV can affect the antigen presentation to suppress adaptive immune response after invading dendritic cells (DCs) (22, 23). In the meanwhile, multiple ASFV genes including *E183L, A179L, A224L, EP153R, DP71L, A238L,* and *E66L* regulate cell apoptosis and autophagy to assist host immune escape (24–26).

While ASFV-host protein-protein interaction (PPI) plays key roles in ASFV invading, replication, and immune escape (24, 27–29), only a few ASFV-host complex structures have been resolved. The ASFV-host PPI landscape, especially for ASFV-host immune pathways interaction, remains largely unknown, impeding the understanding of the exact mechanism of ASFV in host immune escape and vaccine development. Therefore, the aim of this study is to systematically delineate the PPI network between ASFV and host immune pathways, as well as ASFV-ASFV PPI network by the recombination-based library vs library high-throughput yeast two-hybrid (RLL-Y2H) screening system (30). Based on the network, we further explore the ASFV-host PPI difference between domestic pigs and warthogs. Our work will provide substantial resource of ASFV-host immune protein interactome and pave the way to explore the function of ASFV proteins in immune response modulation. It will also contribute to understanding viral pathogenesis and developing ASFV-resistant transgenic pigs.

## RESULTS

### Construction of the PPI network between ASFV and host immune pathways

**Fig 1:**
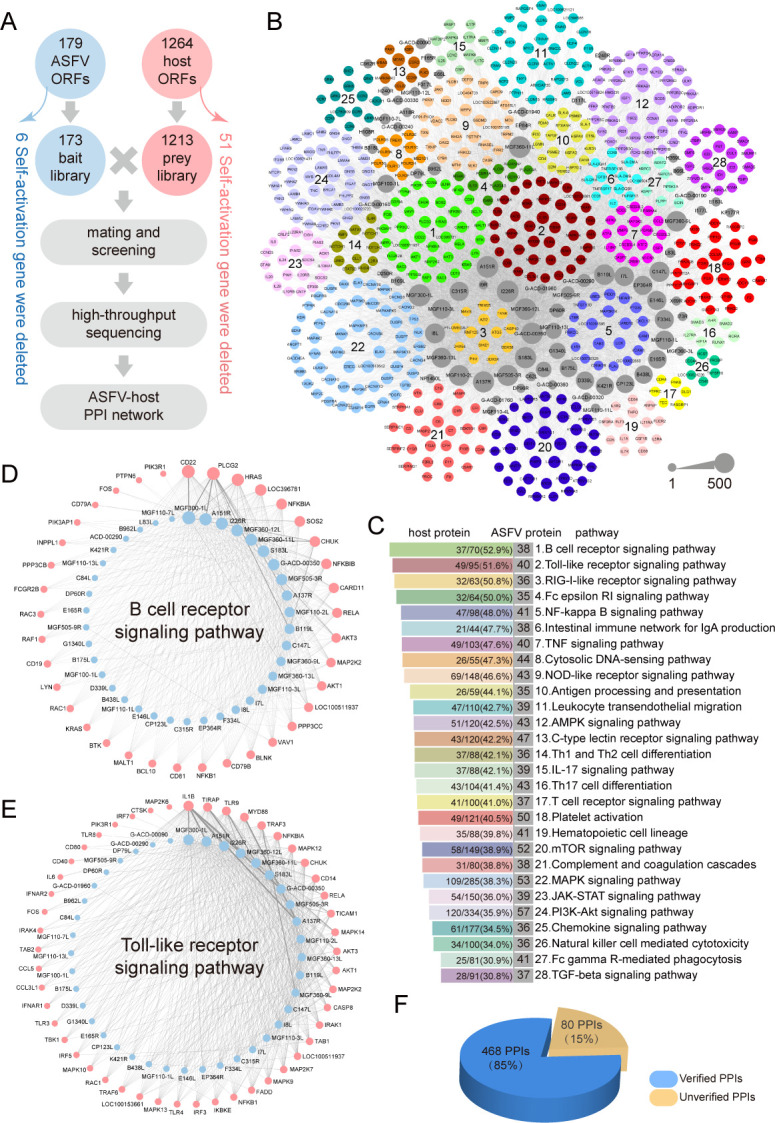
Construction of the PPI network between ASFV and host immune pathways. (**A**) Flowchart of delineating the PPI network between ASFV and host immune pathways by RLL-Y2H. A total of 179 ASFV genes and 1,264 host immune-related genes were cloned and transformed into yeast Y2HGold and Y187 strains, respectively. Six genes from ASFV bait library and 51 genes from host prey library were removed after self-activation screening. The ASFV bait library and host prey library were mated and screened and then subjected to high-throughput sequencing. Based on sequencing data, ASFV-host PPI network was delineated. (**B**) The comprehensive PPI network between ASFV and host immune pathways. Detailed PPI data were listed in Table S2. Gray and color nodes, respectively, represent ASFV proteins and host proteins from 28 distinct immune pathways, in which node size of each protein is proportional to its PPI counts. Pathways enriched with more PPIs were allocated more in center, and ASFV proteins were arranged close to their most enriched pathway. (**C**) Pathway enrichment ranking for 28 immune pathways, of which detailed results were listed in Table S2. (**D and E**) The ASFV-host PPI sub-network of B cell receptor signaling pathway (**D**) and Toll-like receptor signaling pathway (**E**). The blue and pink nodes, respectively, represent ASFV and host proteins, and node size indicates protein PPI counts. Detailed PPI information can be found in Table S2. (**F**) Verification of the PPIs between ASFV and host proteins by Y2H assay. A total of 548 PPIs were re-tested, of which 468 PPIs (85%) were confirmed.

To systematically investigate the interaction network between ASFV and host immune pathway proteins, we employed recombination-based library vs library high-throughput yeast two-hybrid (RLL-Y2H) screening system (30), to systematically screen the potential protein-protein interactions (PPIs). First, 179 ASFV genes were, respectively, cloned into RLL-Y2H bait library vector (Fig. 1A; Table S1). Meanwhile, based on 28 immune pathways from Kyoto Encyclopedia of Genes and Genomes (KEGG) database, 1,264 immune-related genes of *Sus scrofa* were, respectively, cloned into RLL-Y2H prey library vector (Fig. 1A; Table S1). Both libraries were then subjected to self-activation screening, which removed 6 genes from ASFV bait library and 51 genes from host prey library. Subsequently, the bait library and prey library were mated for positive PPIs screening, followed by high-throughput sequencing (Fig. 1A; Fig. S1A through D). After sequencing data analysis, we delineated a comprehensive PPI network containing 8,946 PPIs between 77 ASFV proteins (gray nodes) and 590 host immune pathway proteins (different color nodes) (Fig. 1B; Table S2).

This comprehensive PPI network demonstrated extensive PPIs between ASFV proteins and host proteins from various immune pathways, in which the node size of ASFV and host proteins is in proportion to the PPI counts level (Fig. 1B; Table S2). The immune pathways in the global network were ranked according to the ratio of the number of proteins interacted with ASFV proteins to the number of the whole pathway proteins based on the KEGG pathway enrichment analysis (Fig. 1C; Table S2). Among 28 host immune pathways, the top five enriched pathways were B cell receptor signaling pathway, Toll-like receptor signaling pathway, RIG-I-like receptor signaling pathway, Fc epsilon RI signaling pathway, as well as NF-κB signaling pathway (Fig. 1C). The pathways enriched with more PPIs were allocated more in the center of the whole network. Meanwhile, we also performed pathway enrichment analysis for each ASFV interacting proteins. The locations of ASFV proteins were then arranged close to their most enriched pathway (Fig. 1B; Table S2).

The comprehensive ASFV-host PPI landscape revealed that the ASFV proteins showing obviously higher count of PPIs (indicated with larger gray nodes) with host were attributed by virulent proteins (A151R, I226R, B119L, A137R, MGF360-9L, MGF360-12L, MGF360-13L, MGF505-3R, etc.), replication and transcription-related proteins (C147L, C315R, F334L, E165R, D339L), structural protein (B438L), and some unknown function proteins (MGF300-1L, S183L, I8L, I7L, etc.) (Fig. 1B; Table S2). Of note, RIG-I-like receptor signaling pathway and NF-κB signaling pathway are densely surrounded by ASFV proteins with higher PPI counts (above 100) (Fig. 1B; Table S2). Meanwhile, another group of ASFV proteins (total 16 proteins, most of their function are unknown) with relative lower PPI counts (below 5) was enriched closer to Cytosolic DNA-sensing pathway and C-type lectin receptor signaling pathway (Fig. 1B; Table S2). The intensive interaction between ASFV proteins and these four innate immune pathways suggested that ASFV proteins can systematically disturb innate immunity function.

Next, we deciphered the ASFV-host PPI sub-networks of 28 different immune pathways to further explore the corresponding functions (Table S3). The top eight pathways PPI sub-networks were then individually displayed in Fig. 1D and E; Fig. S2A to F. Of note, more PPIs were formed between host and ASFV virulent proteins, such as A151R, I226R, B119L, A137R, and MGF360-12L (Fig. 1D and E; Fig. S2A through F). Among the PPI sub-networks for top eight pathways, IFN-I induction-associated proteins including NFKB1A, NFKB1B, CHUK, RELA, and TRAF3 were the primary target proteins by ASFV proteins (Fig. 1D and E; Fig. S2A through F). To verify the reliability of PPI between ASFV and host proteins revealed by RLL-Y2H, we randomly re-tested the PPIs between ASFV and host proteins by classical point-to-point Y2H. From all re-tested 548 PPIs, a total of 468 PPIs (85%) were confirmed (Fig. 1F; Fig. S3; Table S2). Of note, there was no significant difference in the positive rate between different PPI frequencies (Table S2), suggesting that both low- and high-frequency PPIs were reliable.

### Construction of the PPI network of ASFV proteins by RLL-Y2H

**Fig 2:**
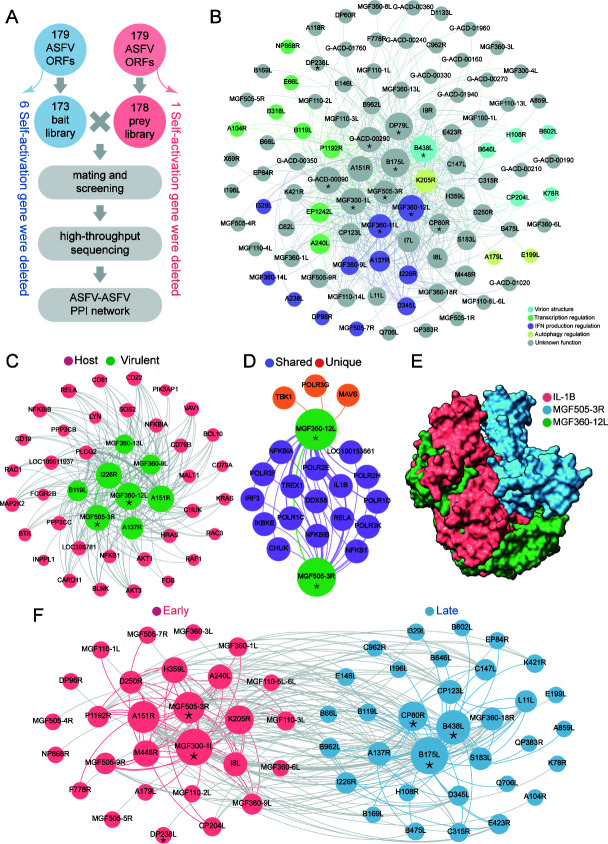
Construction of the PPI network of ASFV proteins. (**A**) Flowchart of delineating the PPI network of ASFV proteins by RLL-Y2H. 179 ASFV genes were cloned and transformed into yeast Y2HGold and Y187 strains, respectively. Six genes from ASFV bait library and one gene from ASFV prey library were removed after self-activation screening. The ASFV bait library and ASFV prey library were mated and screened and then subjected to high-throughput sequencing. PPI network of ASFV proteins was delineated finally. (**B**) The comprehensive PPI network of ASFV proteins. Detailed PPI information was listed in Table S4. (**C**) PPI sub-network of ASFV virulent proteins with host B cell receptor signaling pathway. Green nodes, ASFV virulent proteins; green lines, PPIs between ASFV virulent proteins; pink nodes, host B cell receptor signaling pathway proteins; gray lines, PPIs between ASFV virulent proteins and host B cell receptor signaling pathway proteins. (**D**) The PPI sub-network of MGF505-3R and MGF360-12L with host proteins. Orange nodes, unique host proteins interacting with ASFV MGF360-12L; orange lines, PPIs between MGF360-12L and unique host proteins; green nodes, MGF505-3R and MGF360-12L; green lines, PPIs between MGF360-12L and MGF505-3R; purple nodes, host proteins interacting with both MGF505-3R and MGF360-12L; purple lines, PPIs of both MGF505-3R and MGF360-12L with host proteins. (**E**) 3D structures of protein interaction complex between MGF505-3R, MGF360-12L, and IL-1β by AlphaFold2 simulation. The interaction details were listed in Table S6. (**F**) The PPI sub-networks of ASFV early and late expressed proteins. Pink and blue nodes, respectively, represent early and late proteins. Pink, blue, and gray lines, respectively, correspond to early-early PPIs, late-late PPIs, and early-late PPIs. The PPI details were listed in Table S7. The node size indicates PPI count, and the connection line thickness is in proportion to the interaction frequency.

The large and structurally complex genome of ASFV encodes more than 150 genes, which may form PPI complex with each other to perform functions during invading, replication, immunoregulation, and virion packaging. To decipher the PPI network of ASFV proteins, we further constructed ASFV prey library containing 178 ASFV genes after removing one self-activation gene from a total of 179 ASFV genes and mated the ASFV prey library with ASFV bait library, followed by positive clone screening and high-throughput sequencing (Fig. 2A). Finally, we delineated the whole ASFV PPI network containing 591 PPIs between 98 ASFV proteins (Fig. 2B; Table S4). Proteins were displayed according to their function such as virion structure, replication and transcription regulation, IFN production regulation, autophagy regulation, and unknown function. Interestingly, we observed that several proteins (B438L, MGF360-11L, MGF360-12L, etc.) exhibit self-interaction (marked with *), among which B438L has been reported to form pentamer in the capsid (31, 32).

Considering the important function of ASFV virulent proteins during infection, we further explored the comprehensive PPI network between ASFV virulent proteins (12, 33–44) and host proteins. To this end, we extracted ASFV virulent proteins PPI sub-network from ASFV-ASFV PPI network and integrated it into the ASFV-host PPI network (Table S5). The PPI pathway enrichment ranking for ASFV virulent proteins was listed in Table S5, in which the most enriched pathway was B cell receptor signaling pathway. Fig. 2C demonstrated the ASFV virulent proteins PPI sub-network in the content of ASFV-host B cell receptor signaling pathway PPI network. Furthermore, we illustrated the PPI sub-network between two interactive virulent proteins (MGF360-12L and MGF505-3R) with host proteins. MGF360-12L and MGF505-3R interact with each other and several common host protein partners (purple color) and specific host proteins (orange color), respectively (Fig. 2D). Finally, we elucidated the 3D structure of protein interaction complex between MGF360-12L, MGF505-3R, and IL-1β by AlphaFold2 simulation (Fig. 2E; Table S6). Moreover, in regard to the distinct functions of ASFV early and late expressed proteins (45–48), we also mapped the PPI network of ASFV early and late expressed proteins (Fig. 2F; Table S7).

### Difference of ASFV-host PPI between domestic pigs and warthogs

**Fig 3:**
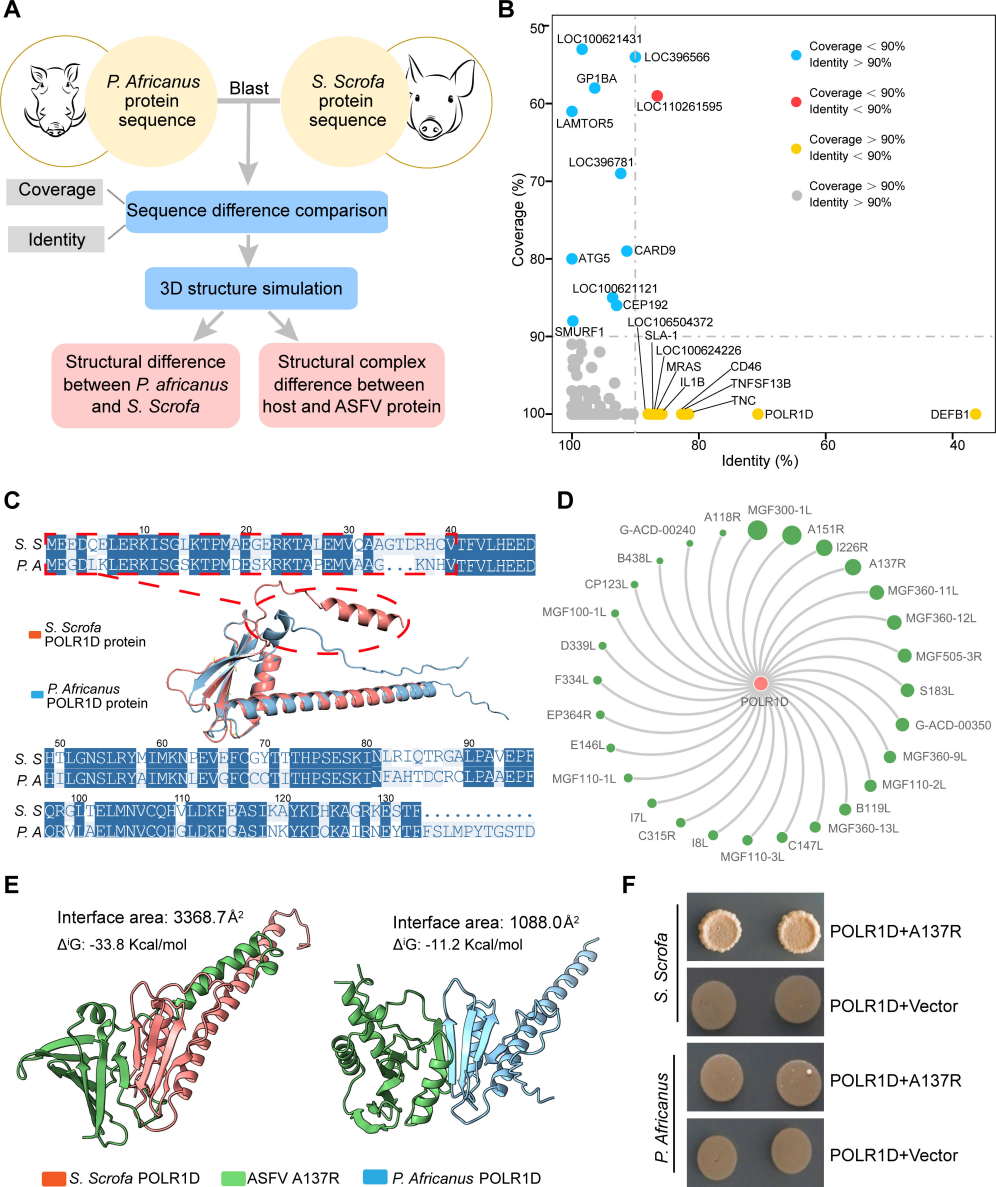
Difference of ASFV-host PPI between domestic pigs and warthogs. (**A**) Flowchart of difference analysis of ASFV-host PPI between *S. scrofa* and *P. africanus*. (**B**) Amino acid (aa) sequences of all 590 host immune proteins in ASFV-host PPI network from *S. scrofa* and *P. africanus* were aligned by BLASTp. The alignment results were listed in Table S8. (**C**) The aa sequence and structural differences of *S. scrofa* and *P. africanus* POLR1D proteins. N terminal 40 aa were highlighted by red line to indicate the corresponding α-helix in *S. scrofa* POLR1D but not in *P. africanus* POLR1D. (**D**) The PPI sub-network between *S. scrofa* POLR1D and ASFV proteins. Green nodes represent ASFV proteins, and pink node represents *S. scrofa* POLR1D protein. (**E**) The 3D interactions simulation of *S. scrofa* POLR1D and *P. africanus* POLR1D with ASFV A137R protein. Interface areas and the solvation-free energy gain upon formation of the interface (Δ^i^G) for each simulated complex were indicated. (**F**) Verification of the PPIs between *S. scrofa* and *P. africanus* POLR1D with ASFV A137R by Y2H assay. Three independent experiments were performed with three technical replicates. *S.S* and *S. scrofa*, *Sus scrofa; P.A* and *P. africanus*, *Phacochoerus africanus*.

Considering the dramatically different clinical symptoms of ASF between domestic pigs (*Sus scrofa, S. scrofa*) and warthogs (*Phacochoerus Africanus, P. africanus*) (4, 5), we tried to investigate the difference of ASFV-host PPI between domestic pigs and warthogs which may underlie the possible mechanism for the pathogenicity discrepancy. To this end, we aligned the protein sequences of all 590 host immune proteins from the ASFV-host PPI network between domestic pigs and warthogs by amino acids sequence analysis (Fig. 3A; Table S8). As shown in Fig. 3B, 569 of 590 proteins (gray nodes) shared high homology between two species according to BLASTp alignment. Since low coverage of sequence is less reliable for interspecies difference analysis, we selected the proteins showing high coverage but low identity (coverage >90% and identity <90%, yellow nodes in Fig. 3B) as potential candidates (DEFB1, POLR1D, TNC, TNFSF13B, CD46, IL-1β, MRAS, LOC100624226, SLA-1, LOC106504372) for further difference analysis of ASFV-host PPI between *S. scrofa* and *P. africanus*.

Next, we used *POLR1D*, a STING-related gene (49) which showed high-sequence divergence between *S. scrofa* and *P. africanus* as an example to further elucidate the distinction of ASFV-host PPI between two species. First, the 3D structure of POLR1D from *S. scrofa* and *P. africanus* was simulated by AlphaFold2, respectively. The details about the sequence and structural differences of POLR1D between *S. scrofa* and *P. africanus* were illustrated in Fig. 3C. There is an obvious α-helix formed by 40 aa at the N terminal of *S. scrofa* POLR1D, while this structure does not exist in *P. africanus* POLR1D. As the difference of these two orthologous proteins may influence their interaction with ASFV proteins, we extracted the PPI sub-network between POLR1D and ASFV proteins from the whole PPI network for further analysis (Fig. 3D).

Among the POLR1D interaction partners, A137R, a late-expressed viral structural protein, has been reported as a negative regulator of the cGAS-STING-mediated IFN-β signaling pathway (12, 50). Thus, we simulated and compared the 3D interaction of *S. scrofa* POLR1D and *P. africanus* POLR1D with ASFV A137R protein by AlphaFold2, respectively. As shown in Fig. 3E, there is a tight interaction between *S. scrofa* POLR1D and ASFV A137R, while the interaction between *P. africanus* POLR1D and ASFV A137R protein showed a much looser interaction. ASFV A137R and *S. scrofa* POLR1D formed more interface areas (3,368.7 Å^2^) than that with *P. africanus* POLR1D (1,088.0 Å^2^). The solvation-free energy gain upon formation of the interface (Δ^i^G) for A137R with *S. scrofa* POLR1D (−33.8 Kcal/mol) was also greater than that with *P. africanus* POLR1D (−11.2 Kcal/mol) (Fig. 3E). Higher values for both interface areas and Δ^i^G indicated the tighter interaction between *S. scrofa* POLR1D and ASFV A137R. To further compare the interaction between *S. scrofa* POLR1D and *P. africanus* POLR1D with ASFV A137R, we synthesized *P. africanus POLR1D* gene (Table S8) and transformed *S. scrofa* and *P. africanus POLR1D* genes together with *A137R* into classical Y2H system, respectively. In line with AlphaFold2 simulation, our Y2H data demonstrated that ASFV A137R interacted with *S. scrofa* POLR1D but not *P. africanus* POLR1D (Fig. 3F).

### Screening of ASFV proteins interfering with cGAS-STING pathway based on PPI network

**Fig 4:**
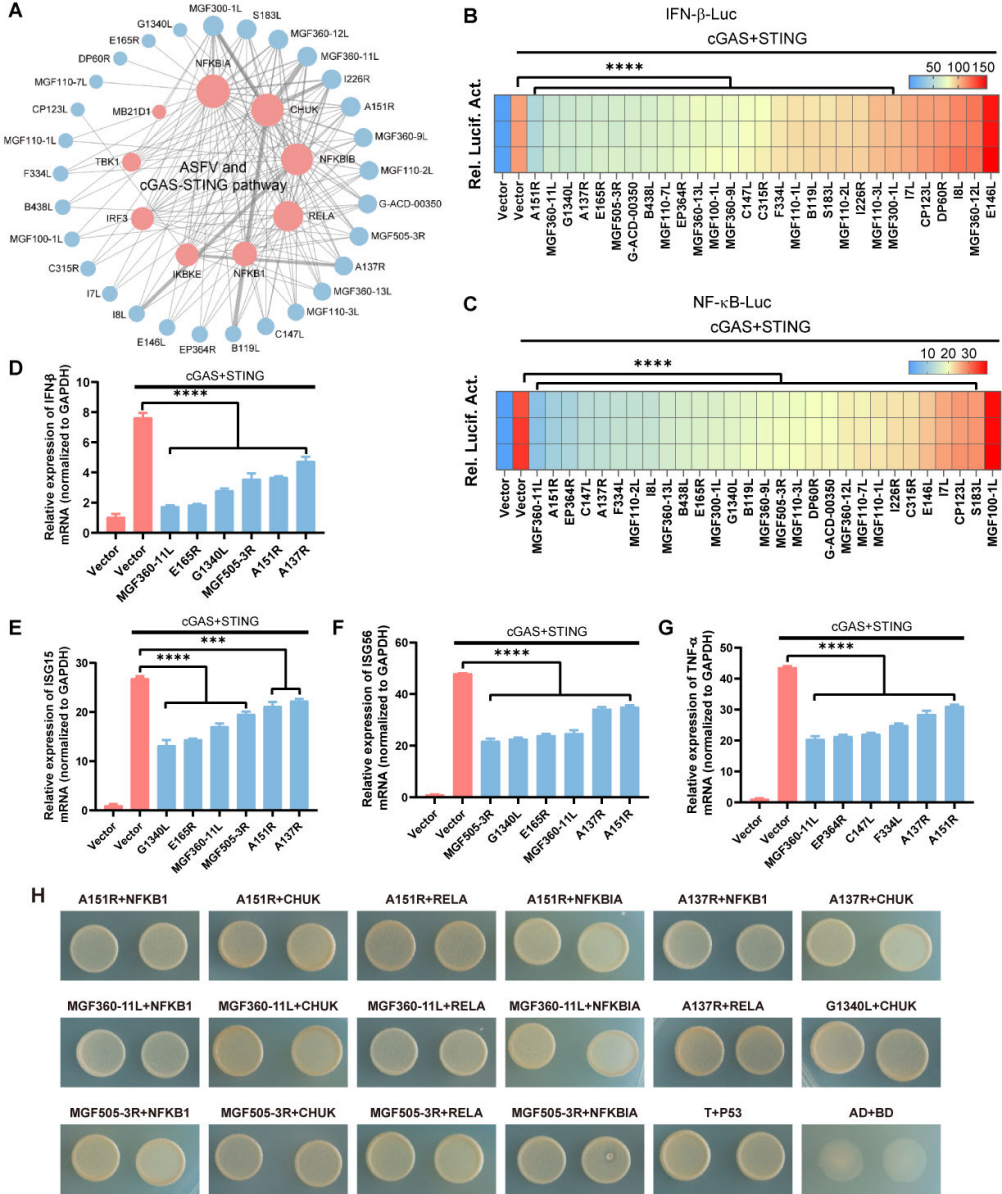
Screening of ASFV proteins interfering with cGAS-STING pathway based on PPI network. (**A**) The PPI sub-network between ASFV and cGAS-STING pathway proteins. Blue nodes, ASFV proteins; pink nodes, host cGAS-STING pathway proteins. (**B and C**) HEK293T cells were transfected with IFN-β-Luc (human) (**B**) or NF-κB-Luc (human) (**C**) reporter, pRL-TK reporter, HA-cGAS (human), and HA-STING (human), together with different plasmid expressing HA-ASFV, respectively. At 24 hpt, Luc activities were then evaluated through the dual-luciferase assay. (**D–F**) HEK293T cells were transfected with HA-cGAS (human) and HA-STING (human), together with ASFV gene eukaryotic expression plasmid (HA-A151R, MGF360-11L, G1340L, A137R, E165R, or MGF505-3R) for 24 h. The mRNA levels of IFN-β (**D**), ISG15 (**E**), and ISG56 (**F**) in the HEK293T cells were analyzed by qRT-PCR assay. (**G**) HEK293T cells were transfected with HA-cGAS (human) and HA-STING (human), together with ASFV gene eukaryotic expression plasmid (HA-MGF360-11L, A151R, EP364R, C147L, A137R, or F334L) for 24 h. The mRNA levels of TNF-α in the HEK293T cells were analyzed by qRT-PCR assay. (**H**) Verification of the PPIs by Y2H assay. ****P < 0.0001; ***P < 0.001 (one-way ANOVA). Data are representative of three independent experiments with three biological replicates (mean ± SEM).

Next, we aimed to explore the potential functions of ASFV-host PPI network. As a cytoplasmic dsDNA virus, ASFV can intensely activate DNA-sensing cGAS-STING pathway (51). Thus, we tried to elucidate the potential effect of ASFV proteins in ASFV-host PPI network on cGAS-STING pathway. To this end, the sub-network between ASFV and host cGAS-STING pathway was extracted from the whole PPI network (Fig. 4A). In this sub-network, 29 ASFV proteins (blue nodes) interact with 9 host proteins (pink nodes) involved in cGAS-STING pathway. To explore which ASFV protein in this network may perturb cGAS-STING-induced immune response, we performed an unbiased screening of all 29 ASFV proteins on the inhibition of IFN-β and NF-κB promoter activity. To this end, we co-transfected 29 ASFV genes individually with IFN-β-luc or NF-κB-luc, HA-cGAS (human), HA-STING (human), and internal control pRL-TK plasmids into HEK293T cells and evaluated activation of the IFN-β and NF-κB promoter according to the fluorescence intensity, respectively. Of note, 23 of 29 ASFV genes including *A151R*, *MGF360-11L*, *G1340L*, and *A137R* could significantly inhibit cGAS-STING induced IFN-β promoter activation (Fig. 4B) and 28 of 29 ASFV genes including *MGF360-11L*, *A151R*, *EP364R*, and *C147L* could significantly inhibit cGAS-STING induced NF-κB promoter activation (Fig. 4C). Meanwhile, we confirmed the inhibitory effect of the top 15 ASFV proteins on cGAS-STING pathway using swine cGAS and STING plasmids (Fig. S4A and B). And the data showed that among the 15 ASFV genes we tested, 13 genes (~87%) could inhibit cGAS-STING-induced IFN-β promoter activation and 14 genes (~93%) could inhibit NF-κB promoter activation, which is consistent with our screening result.

Moreover, we performed qRT-PCR to confirm the function of the ASFV genes (*A151R, MGF360-11L, E165R, G1340L, MGF505-3R,* and *A137R*) with prominent inhibitory effect on IFN-β production. As shown in Fig. 4D through F; Fig. S4C to E, these genes can, indeed, significantly decrease the mRNA expression level of *IFN-*β as well as two IFN stimulated genes *ISG15* and *ISG56*. Meanwhile, the inhibitory function of the ASFV genes (*K421R, MGF360-11L, EP364R, C147L, A151R,* and *A137R*) with prominent inhibitory effect on NF-κB production was also confirmed, as indicated by the markedly reduced level of *TNF-*α mRNA upon transfection of these ASFV genes (Fig. 4G; Fig. S4F). Furthermore, we performed Y2H assay to further validate the PPIs between these ASFV proteins and host cGAS-STING pathway (Fig. 4H). Together, our data suggest that these ASFV proteins may inhibit the production of IFN-β, NF-κB and the subsequent anti-viral factors by targeting the host cGAS-STING pathway.

## DISCUSSION

Deciphering of the interactome between ASFV and host immune pathway proteins can provide vast resource for the functional exploration of ASFV proteins and the understanding of ASF pathogenesis. In this study, we deciphered the comprehensive PPI landscape between ASFV and host immune pathways via RLL-Y2H screening. Our data suggest that in the ASFV-host interactome, the ASFV virulent proteins were the primary contributors and intensively interact with host innate immune pathways. Most known ASFV proteins have been reported to interfere with innate immune response including IFN-I and NF-κB production (10–16). In this scenario, we observed that the IFN-I and NF-κB production pathways are the primary targets of ASFV proteins, suggesting ASFV may dampen IFN-I and NF-κB production via PPI. Meanwhile, B cell receptor signaling pathway proteins are also extensively interacted with ASFV proteins especially virulent proteins, implying ASFV may inhibit antibody production. In addition, ASFV proteins also interact with PI3K-Akt signaling pathway, MAPK signaling pathway, and mTOR signaling pathway, which are essential for cell inflammation, stress response, metabolism, autophagy, and apoptosis. It would be of great importance to further investigate the roles of these ASFV proteins in counteracting various signaling pathways.

Our study also revealed the ASFV-ASFV PPI network, in which we identified several self-interaction ASFV proteins such as B438L, DP79L, B175L, MGF300-1L, MGF360-11L, MGF360-12L, and MGF505-3R, suggesting they may form dimer or multimers to perform function. Through combining ASFV-ASFV PPI network with ASFV-host PPI network, we delineated a detailed interactome containing intra- and inter-PPI between ASFV virulent proteins with host immune proteins, such as intra-connection of MGF505-3R with MGF360-12L and their inter-connection with host proteins. With the rapid advance of AlphaFold2, the detailed structure of ASFV and host proteins complex can be further resolved. This integrated analysis of interactome and structure will contribute to comprehensively explore the protein interaction of ASFV-host and their function during pathogenies.

RLL-Y2H system can screen the direct PPIs but may miss the indirect PPIs or PPI complex that require more than two members. Thus, when two viral proteins interact with each other to form complex with the host proteins but either of them alone is insufficient for interaction with the host protein, our screen system cannot identify this kind of PPIs. Nevertheless, even in this scenario, our ASFV-ASFV PPI network can provide important information for such ASFV-host protein complex relying on certain ASFV proteins interaction.

While ASFV causes high mortality for domestic pigs, it causes much mild clinical symptoms for bushpigs and warthogs. The sequence and structure difference as well as PPI discrepancy of orthologous proteins between these animals may attribute to this distinct pathogenesis. Our analysis revealed dozens of candidate proteins with significant sequence difference between the domestic pigs and warthogs’ orthologous proteins from the 590 host proteins in the PPI network. Moreover, different simulated 3D structures for POLR1D and the interaction with ASFV protein were observed between two species. The structure analysis of the protein complex of ASFV A137R and host POLR1D proteins of domestic pigs and warthogs revealed an obvious different interaction strength between these proteins, which was also validated by Y2H assay. It has been reported that A137R can inhibit cGAS-STING-mediated IFN-β signaling pathway (12, 50) and *POLR1D* is a STING-related gene. In line with this, our dual-luciferase reporter assay and qRT-PCR assay demonstrated that A137R can, indeed, inhibit the cGAS-STING-induced IFN-β and NF-κB promoter activity and the mRNA expression of *IFN-*β, *ISG15*, *ISG56*, and *TNF-*α. It will be of great interest to further investigate whether the inhibition of A137R on cGAS-STING pathway is mediated by its interaction with POLR1D and whether the interaction discrepancy is responsible for the susceptibility to ASFV between domestic pigs and warthogs. As POLR1D is a component of RNA polymerase I and RNA polymerase III complexes, the interaction of ASFV protein with POLR1D may also affect transcription instead of immune signaling. Together, these data suggested that this PPI discrepancy might be one of the reasons underlying the different clinical symptoms between ASFV susceptive and resistant species.

It has been reported that ASFV proteins can disturb cGAS-STING pathway-mediated IFN-I and NF-κB production. Based on our ASFV-host cGAS-STING pathway PPI sub-network, we screened the inhibitory effect of ASFV proteins in this sub-network on cGAS-STING pathway-mediated IFN-β and NF-κB promoter activity. Our data revealed that 29 ASFV proteins can interact with cGAS-STING pathway proteins, most of which can influence the cGAS-STING stimulated IFN-β and NF-κB promoter activity. It suggested that ASFV utilized multiple proteins to perturb cGAS-STING pathway, which may increase their effect. Among these proteins, the regulatory effect of A137R, MGF360-11L, and MGF505-3R has been previously reported (12, 16, 17), supporting the reliability of our analysis. While these data are still at *in vitro* level, the modulatory effect on cGAS-STING pathway and PPI with the host proteins in this pathway provide an ample resource for further functional study of ASFV pathogenesis. Here, we performed these screening assays in HEK293T cells but not porcine cells for two reasons. On one hand, the transfection efficiency in porcine cells is low. On the other hand, previous studies have shown that the inhibition of ASFV proteins on cGAS-STING pathway in HEK293T cells could be recapitulated in porcine cells such as porcine alveolar macrophages (14, 19, 52). However, to further study the functional mechanism of these ASFV proteins, it would be important to validate their inhibitory function on cGAS-STING pathway in porcine cells and *in vivo.*

In conclusion, our study provided a substantial resource of ASFV-ASFV and ASFV*-*host protein interactome, which may greatly facilitate the functional study of ASFV proteins and the pathogenesis of ASF. The systematical comparative analysis of PPI protein sequences and their discrepancy in the PPI with ASFV proteins between *S. scrofa* and *P. africanus* will shed light on the distinct ASF pathogenesis in different species and may pave the way for generation of ASFV-resistant transgenic pigs. Finally, our data may also provide important clues on potential targets for the development of vaccines and anti-viral strategies for ASFV control.

## MATERIALS AND METHODS

### RLL-Y2H bait and prey libraries construction

To investigate the PPI network between ASFV and host immune proteins as well as the PPI network of ASFV proteins, respectively, three libraries including ASFV bait library, host prey library, and ASFV prey library were constructed based on RLL-Y2H system (30). For construction of ASFV bait and prey libraries, the genomic DNA of ASFV HLJ/18 isolate (GenBank accession number: MK333180.1) was used as a template (53). A total of 179 ASFV genes were amplified and cloned into pmBD and pmAD vector, respectively, for bait and prey library construction. For ASFV genes over 1,500 nt (28 genes), only 1,500 nt sequence at the C terminal was amplified and cloned. Briefly, PCR products with homologous arms were together transformed with linearized pmBD or pmAD into yeast strain Y2Hgold (for bait library) or Y187 (for prey library) and then, respectively, plated on SD/-T or SD/-L selective plates. To confirm the correct integration of ASFV PCR products into vectors, the growing colonies were amplified and subjected to Sanger sequencing or plasmid digestion. Then, positive colonies for 179 genes of ASFV bait library and prey library were separately mated with Y187 harboring empty pmAD or Y2Hgold strain harboring empty pmBD vector and plated onto SD/-LT and SD/-AHLT selective plates to test their self-activation. Those not showing any self-activation were pooled as ASFV bait or prey library. Gene sequences and primers used for ASFV bait and prey libraries construction and self-activation genes were listed in Table S1.

For constructing host prey library, total RNAs were isolated from domestic pigs (*Sus scrofa*) liver, spleen, lung, and brain tissues using Trizol reagent (15596026, Thermo Fisher Scientific, Waltham, MA, USA) according to the manufacturer’s instructions. Total RNAs were processed directly to synthesize cDNA with ABScript III RT Master Mix (RK20429, ABclonal Biotech Co, Wuhan, China) following the manufacturer’s instructions. Then, a total of 1,264 genes were amplified from the cDNA and cloned into pmAD vector by transformation and recombination into yeast strain Y187. For genes over 1,500 nt (532 genes), only 1,500 nt sequence at the C terminal were amplified and cloned. All 1,264 host genes belong to 28 immune pathways according to KEGG database, including hematopoietic cell lineage, complement and coagulation cascades, platelet activation, Toll-like receptor signaling pathway, NOD-like receptor signaling pathway, RIG-I-like receptor signaling pathway, cytosolic DNA-sensing pathway, C-type lectin receptor signaling pathway, natural killer cell-mediated cytotoxicity, antigen processing and presentation, T cell receptor signaling pathway, Th1 and Th2 cell differentiation, Th17 cell differentiation, IL-17 signaling pathway, B cell receptor signaling pathway, Fc epsilon RI signaling pathway, Fc gamma R-mediated phagocytosis, leukocyte transendothelial migration, intestinal immune network for IgA production, chemokine signaling pathway, MAPK signaling pathway, TGF-β signaling pathway, JAK-STAT signaling pathway, NF-κB signaling pathway, TNF signaling pathway, PI3K-Akt signaling pathway, AMPK signaling pathway, and mTOR signaling pathway. Then, positive colonies for 1,264 genes were separately mated with Y2Hgold strain harboring empty pmBD vector and plated onto SD/-AH and SD/-AHLT selective plates to test their self-activation. Those not showing any self-activation were pooled as host prey library. Gene sequences and primers used for host prey library construction and self-activation genes were listed in Table S1.

### RLL-Y2H screening, high-throughput sequencing, and data analysis

The bait and prey libraries were mated for 24 h. Then, yeast cells were collected and resuspended in 50 mL 0.9% NaCl and then plated onto SD/-AHLT selective plates for ~7 days growth. All positive colonies from SD/-AHLT plates were pooled to isolate the integrated plasmids using the yeast plasmid kit (D3376, OMEGA Bio-Tek, Norcross, GA, USA) according to the manufacturer’s instructions.

The recombined bait and prey fragments were amplified using the isolated integrated plasmids from the pooled positive colonies for 20 cycles. A smear band should be visible on agarose gel (Fig. S1B). Digesting the purified PCR products with *Mme*I (R0637, NEB, Ipswich, MA, USA) yields a ~110 bp target band (Fig. S1C), followed by PAGE gel purification. Next, by annealing overhang NN-nucleotides, we prepared MGI adapter A (5′- GAACGACATGGCTACGATCCGACTTNN-3′ and 5′- AAGTCGGATCGTAGCCATGTCGTTC-3′) and adapter B (5′- AAGTCGGAGGCCAAGCGGTCTTAGG-3′ and 5′-CCTAAGACCGCTTGGCCTCCGACTTNN-3′). The adapters A and B were ligated with purified 110 bp fragments by T4 ligase at 16°C for ~2 h. Then, the ligated products were amplified using sequencing primers [5′-P-GAACGACATGGCTACGA-3′ and 5′-TGTGAGCCAAGGAGTTG (index)TTGTCTTCCTAAGACCGCTTGGCCTCCGACT-3′] to construct high-throughput sequencing library (Fig. S1D), followed by sequencing by MGISEQ-2000 platform (BGI, China). The sequencing data have been uploaded into Gene Expression Omnibus (45) under accession number GSE220585.

For interacting protein pair analysis, the tool RLLNP (https://github.com/thxie/RLLNP) was used to obtain the protein interaction information from RLL-Y2H sequencing raw data. Briefly, the semi-global alignment algorithm was used to scan all the sequencing reads for *Mme*I-ATTL-*Mme*I site “gttggaTAGCGTGCGGGTGCCAGGGCGTGCCCTTGAGTTCTCTCAGTTGGGGGCGTTGACtccgac” and then retained the reads containing about 13 bp DNA flanking both sides of *MmeI*-ATTL-*MmeI* structure for subsequent analysis. We also built the reference libraries based on the genes in bait and prey libraries (173 genes of ASFV bait library, 178 genes of ASFV prey library, and 1,213 genes of host prey library). Next, the trimmed sequencing pairs were aligned against the reference libraries using the unique alignment algorithm in RLLNP. Unique mapping reads with no mismatch and the sequence pairs in which both of reads could be aligned unambiguously with no mismatch were retained and output as tables for the whole ASFV-host PPI and ASFV-ASFV PPI; then, all sub-networks for ASFV-host and ASFV-ASFV PPI were further extracted (Tables S2 to S7). Interaction frequency of an interacting protein pair could be calculated by counting the sequence pairs that could be aligned to them. PPI count for each protein of ASFV and host was calculated as the number of all interactive host and ASFV proteins, respectively. To verify the reliability of ASFV-host PPI, we randomly selected 548 PPIs to re-test their interaction by point-to-point Y2H (Table S2).

Based on these PPI data, ASFV-host and ASFV-ASFV PPI network and sub-network can be drawn and visualized by Cytoscape (V 3.4.0) software (54) and Gephi (V 0.9.2) software (55). In the network, the node size indicates PPI count of the proteins, and the connection line thickness is in proportion to the interaction frequency. To better illustrate ASFV-host PPI network, all pathways were roughly located according to their pathway ranking based on pathway enrichment analysis (Table S2) as higher rankings in the center but lower ones in the border region.

For pathway enrichment analysis, PPI protein number and total protein number of each pathway were counted separately using R statistical environment (https://www.R-project.org/), and the ratios of PPI protein number to total protein number for each pathway were ranked (Tables S2 and S5). Meanwhile, the ASFV proteins in the ASFV-host PPI network were arranged according to their PPI enrichment pathway, meaning more closer to their highest-ranking pathway (Table S2).

### Amino acid sequence alignment analysis of *Sus scrofa* (*S. scrofa*) and *Phacochoerus africanus* (*P. africanus*)

The amino acid sequences of 590 proteins of *S. scrofa* in ASFV-host PPI network were downloaded from KEGG. The data package of amino acid sequences of *P. africanus* proteins was downloaded from NCBI (ROS_Pafr_v1, RefSeq GCF_016906955.1). BLAST (https://blast.ncbi.nlm.nih.gov/Blast.cgi) was used to align the amino acid sequence of orthologous sequence couples between *S. scrofa* and *P. africanus* to align their difference (56). To determine the homologous sequences with the highest difference, all BLAST hits for each aligned sequence couples were filtered by both *E*-value (the number of expected hits of similar quality [score] that could be found just by chance) higher than 10^−5^ and the highest bit scores to finally obtain the highest hit for each couple for 590 host proteins. Protein sequence alignment results for orthologous proteins from *S. scrofa* and *P. africanus* were listed in Table S8. To present the final alignment results, proteins higher than 90% in both identity rate (percentage of identical matches in amino acids) and coverage rate (query of *S. scrofa* coverage per subject of *P. africanus*) were not significant difference between *S. scrofa* and *P. africanus* which showed in gray color. The proteins lower than 90% coverage rate and higher than 90% identity rate were shown in blue color. The proteins lower than both 90% coverage rate and 90% identity rate were shown in red color. Only proteins lower than 90% identity rate but higher than 90% coverage rate were possible candidates for further analysis on the PPI difference with ASFV proteins between *S. scrofa* and *P. africanus*, which showed in yellow color. For each coupled homologous sequences, the sequence difference details including amino acids difference were visualized by DNAMAN (version 6) and 3D structure difference was further illustrated by AlphaFold2 (v.2.1.1) (57).

### 3D protein structure simulation

To simulate the 3D structure of protein interaction between AFSV and host, as well as the 3D structure difference of homologous proteins between *S. scrofa* and *P. africanus*, amino acid sequences of all concerned proteins were imported into AlphaFold2 (v.2.1.1). The parameters for AlphaFold2 were set as --max_template_date = 2020–05-14 --model_preset = monomer to simulate the 3D structure of each protein sequence. For protein interaction simulations, amino acid sequences for interactive proteins were imported into AlphaFold-multimer (58) and run with --max_template_date = 2020-5-14 --model_preset = multimer and Uniprot database and PDB reqres database (two databases were download at Jan 12, 2022). AlphaFold2 software would produce five possible 3D protein structure models for single protein and interactive proteins, of which the highest confidence model would be visualized in ChimeraX (v.1.3) (59).

To present the significant interaction position of simulated 3D structures for interactive proteins, the VDW (van der Waals) overlap parameter was set over 0.40 Å, and interactions between atoms with four or fewer bons would be ignored. The interface of each interaction models was analyzed by PDBePISA (https://www.ebi.ac.uk/msd-srv/prot_int/pistart.html) to obtain the number of interfacing atoms, the number of interfacing residues, and the total solvent accessible surface area in square Angstroms in each structure. Then, the PDBePISA would calculate interfaces area as difference in total accessible surface areas of isolated and interfacing structures divided by two, and the solvation free energy gain upon formation of the interface in kcal/mol.

### ASFV eukaryotic expression plasmid construction

To construct plasmids expressing HA-tagged *MGF300-1L, S183L, MGF360-12L, MGF360-11L, I226R, A151R, MGF360-9L, MGF110-2L, G-ACD-00350, MGF505-3R, A137R, MGF360-13L, MGF110-3L, C147L, B119L, EP364R, E146L, I8L, I7L, C315R, MGF100-1L, B438L, F334L, MGF110-1L, CP123L, MGF110-7L, DP60R, E165R,* and *G1340L*, the 29 ASFV genes were amplified on the basis of the genome of the ASFV HLJ/18 isolate (53) and cloned into eukaryotic expression vector pCAGGS-HA (pHA). All the plasmids constructed were analyzed and verified by DNA sequencing (Shanghai Sangon Biotech, China). The primers used in plasmid construction were listed in Table S9.

### Dual-luciferase reporter assay

HEK293T cells (ATCC CRL-3216) were seeded into 24-well plates (Corning, USA) and grown to 80%–90% confluence in Dulbecco’s modified Eagle’s medium (DMEM) containing 10% fetal bovine serum (FBS), 100 IU/mL penicillin, and 100 µg/mL streptomycin at 37°C with 5% CO_2_. The monolayer cells were co-transfected with reporter plasmids 0.1 µg/well IFN-β-luc (human) or NF-κB-luc (human) plus 0.05 µg/well pRL-TK, with or without 0.1 µg/well pHA-cGAS (human) and 0.1 µg/well pHA-STING (human) plasmids, as well as 0.6 µg/well pHA-ASFV gene or vector control plasmid using Polyethylenimine Linear (43) MW40000 (40816ES03, Yeasen Biotechnology, Shanghai, China). The cells were lysed at 24 hours post transfection (hpt), and the firefly and Renilla luciferase activities in the lysates were measured using a dual luciferase reporter assay kit according to the manufacturer’s protocol (DL101, Vazyme Biotech Co., Nanjing, China). The fold changes were calculated relative to control samples after normalization of firefly luciferase by Renilla luciferase. All experiments were performed in triplicate and repeated at least three times.

### Quantitative real-time PCR (qRT-PCR)

HEK293T cells (ATCC CRL-3216) were seeded into 24-well plates (Corning, USA) and grown to 80%–90% confluence in DMEM containing 10% FBS, 100 IU/mL penicillin, and 100 µg/mL streptomycin at 37°C with 5% CO_2_. The monolayer cells were co-transfected with pHA-ASFV gene or vector control plasmid, pHA-cGAS (human), and pHA-STING (human) plasmids using Polyethylenimine Linear (43) MW40000 (40816ES03, Yeasen Biotechnology, Shanghai, China). To detect *IFN-*β, *ISG15*, *ISG56*, and *TNF-*α mRNA levels, total RNA was extracted 24 hpt using Trizol reagent (15596026, Thermo Fisher Scientific, Waltham, MA, USA), and reverse transcription was performed with ABScript III RT Master Mix for qPCR with gDNA Remover (RK20429, ABclonal Biotech Co, Wuhan, China). Reverse transcription products were amplified with 2× Universal SYBR Green Fast qPCR Mix (RK21203, ABclonal Biotech Co, Wuhan, China) according to the manufacturer’s instructions. All the reactions were triplicated and performed in the QuantStudio5 system (Applied Biosystems, Foster City, CA, USA). The PCR was performed by 30 cycles of denaturation at 94°C for 1 min, annealing at 55°C for 1 min, and extension at 72°C for 1 min. To confirm the specificity of amplification, the PCR products from each primer pair were subjected to a melting curve analysis and electrophoresis in 2% agarose gel. Primers used for qRT-PCR were listed in Table S9. Furthermore, the *Ct* values for each gene amplification were normalized with internal control GAPDH by the 2^–ΔΔ*Ct*^ method. All the qPCR experiments were conducted in duplicates in each experiment, and experiments were replicated at least three times.

### Statistical analysis

The significance of the experimental results was analyzed by using one-way ANOVA with GraphPad Prism v.8 (*P < 0.05; **P < 0.01; ***P < 0.001; ****P < 0.0001; ns indicates no significance). Data were expressed as the mean ± SEM of at least three independent experiments. The results were from one representative experiment.

## Data Availability

The sequencing data have been uploaded into Gene Expression Omnibus under accession number GSE220585.

## SUPPLEMENTAL MATERIAL

Supplemental figures msystems.00471-23-s0001.pdf Fig. S1-S4.

Table S1 msystems.00471-23-s0002.xlsx The primers and gene sequences for the construction of host prey library, ASFV bait library, and ASFV prey library.

Table S2 msystems.00471-23-s0003.xlsx Detailed ASFV-host PPIs between 77 ASFV proteins and 590 host immune pathway proteins.

Table S3 msystems.00471-23-s0004.xlsx Detailed ASFV-host PPIs for 28 sub-networks.

Table S4 msystems.00471-23-s0005.xlsx Detailed ASFV-ASFV PPIs between 98 ASFV proteins.

Table S5 msystems.00471-23-s0006.xlsx Detailed PPIs between ASFV virulent proteins and host.

Table S6 msystems.00471-23-s0007.xlsx Detailed interaction information between MGF505-3R, MGF360-12L, and IL-1B.

Table S7 msystems.00471-23-s0008.xlsx Detailed ASFV-ASFV PPIs for early and late ASFV proteins.

Table S8 msystems.00471-23-s0009.xlsx Protein sequence alignment results of 590 host proteins in the ASFV-host PPI network between domestic pigs (*Sus scrofa*) and warthogs (*Phacochoerus africanus*).

Table S9 msystems.00471-23-s0010.xlsx Primers for ASFV eukaryotic expression plasmids construction and for qRT-PCR.

ASM does not own the copyrights to Supplemental Material that may be linked to, or accessed through, an article. The authors have granted ASM a non-exclusive, world-wide license to publish the Supplemental Material files. Please contact the corresponding author directly for reuse.
