# Supplementary material for: Interactome between ASFV and host immune pathway proteins
Source: mSystems. 2023 Nov 15;8(6):e00471-23. doi: 10.1128/msystems.00471-23 (PMC10734461; doi:10.1128/msystems.00471-23)
Supplement: Supplemental figures — Fig. S1-S4. [file msystems.00471-23-s0001.pdf]

SUPPLEMENTAL MATERIAL

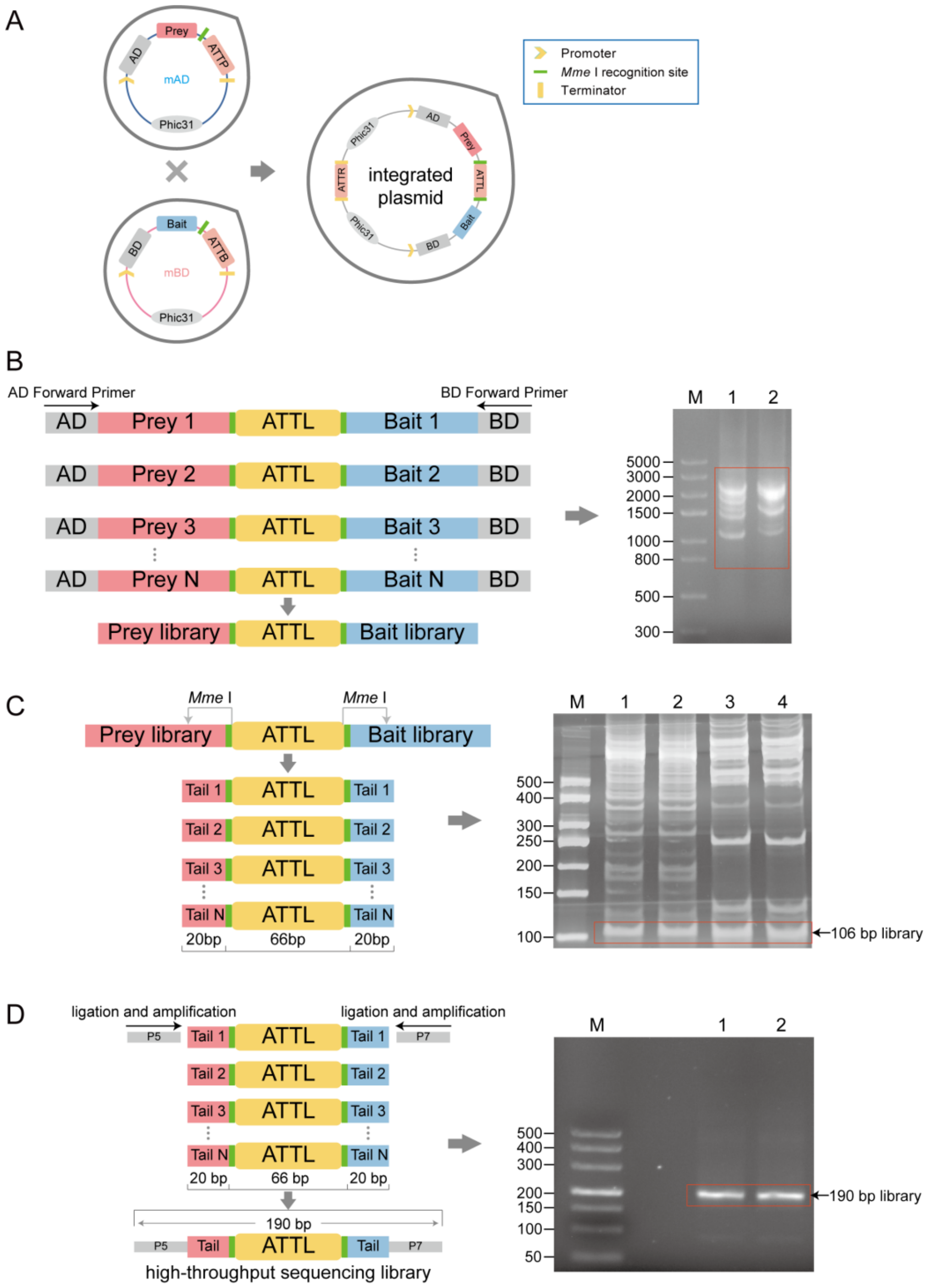

Fig S1. ASFV-host and ASFV-ASFV RLL-Y2H sequencing libraries construction. The recombination of bait and prey plasmids by PhiC31 after mating RLL-Y2H prey and bait libraries. The integrase PhiC31 recognizes ATTB and ATTP sites and integrates the bait and prey plasmids into a big plasmid *in vivo* which unambiguously couples the interacting pair of specific bait and prey genes. (**B**) Amplification of ligated prey and bait fragments. After plasmid isolation from the positive colony pool, the ligated prey

and bait fragments were amplified (left panel). PCR product (marked with red box) of ligated prey and bait fragments were observed on agarose gel electrophoresis (right panel). M, DNA marker; 1, Ligated ASFV bait and host prey fragments; 2, Ligated ASFV bait and ASFV prey fragment. (**C**) Digestion of ligated prey and bait fragments by *Mme*I. Ligated prey and bait fragments were digested by *Mme*I (left panel), then the digested fragments (~110 bp, marked with red box) were observed on agarose gel electrophoresis (right panel). M, DNA marker; 1-2, *Mme*I digestion of PCR product 1 in (B); 3-4, *Mme*I digestion of PCR product 2 in (B). (**D**) Ligation of digested fragments with sequencing adaptors. Digested fragments from C were ligated with sequencing adaptors (P5, P7) to generate sequencing fragments. Then the sequencing fragments were amplified (~190 bp) to finally generate RLL-Y2H sequencing library (left panel). Electrophoresis results of the RLL-Y2H sequencing library (marked with red box) were shown in the right panel. M, DNA marker; 1, ASFV-host sequencing library; 2, ASFV-ASFV sequencing library.

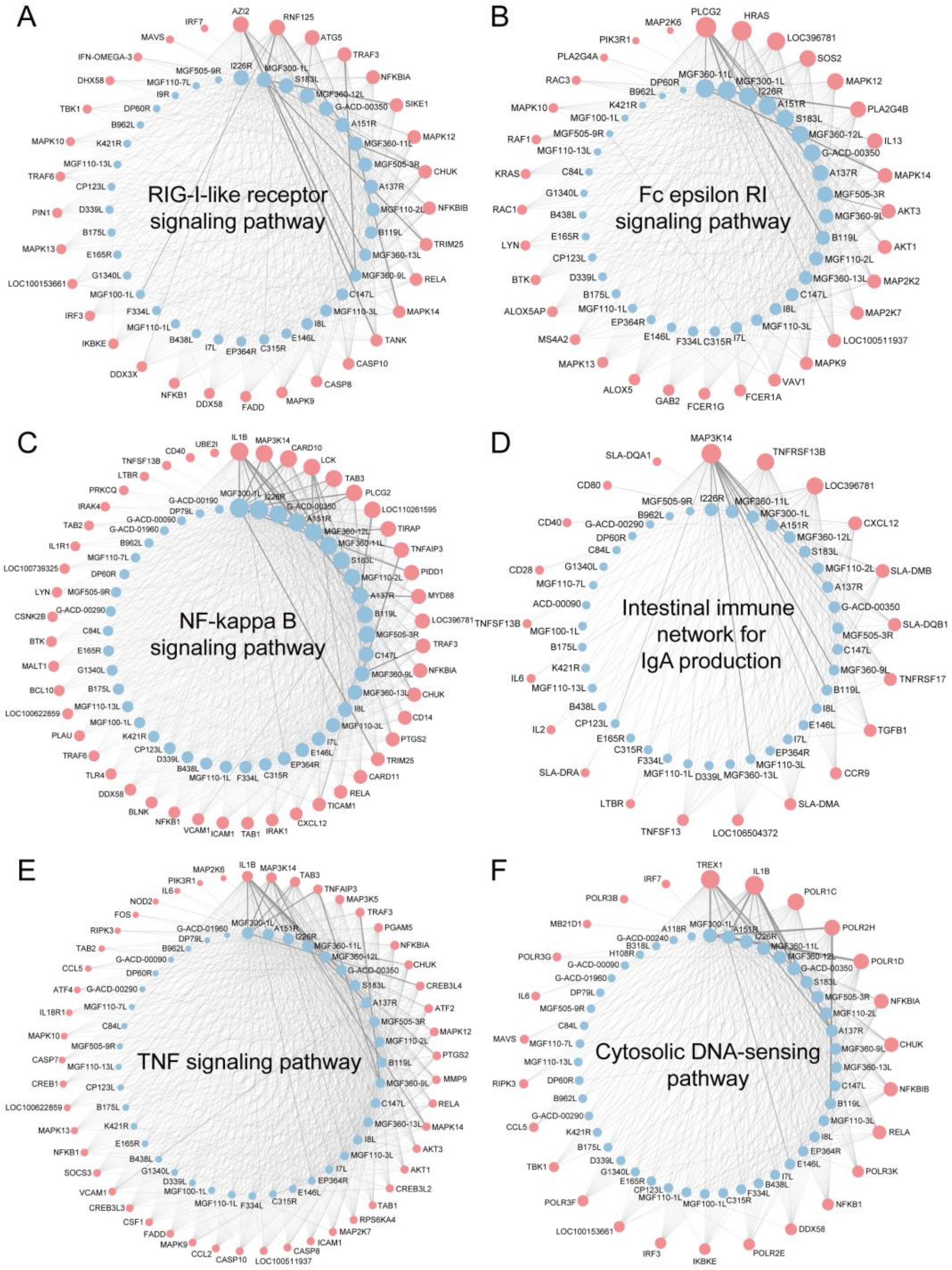

Fig S2. ASFV-host PPI sub-network for immune pathway. (A-F) ASFV-host PPI sub-network for RIG-I-like receptor signaling pathway (A), Fc epsilon RI signaling pathway (B), NF-κB signaling pathway (C), intestinal immune network for IgA production (D), TNF signaling pathway (E), and cytosolic DNA-sensing pathway (F). The 6 immune pathways correspond to KEGG pathway enrichment ranking from 3rd to 8th in Fig1C. Detailed ASFV-host PPI information of each pathway were listed in

Table S3, and the node size for ASFV proteins (blue) and host proteins (pink) is in proportion to the PPI counts of proteins in Table S3.

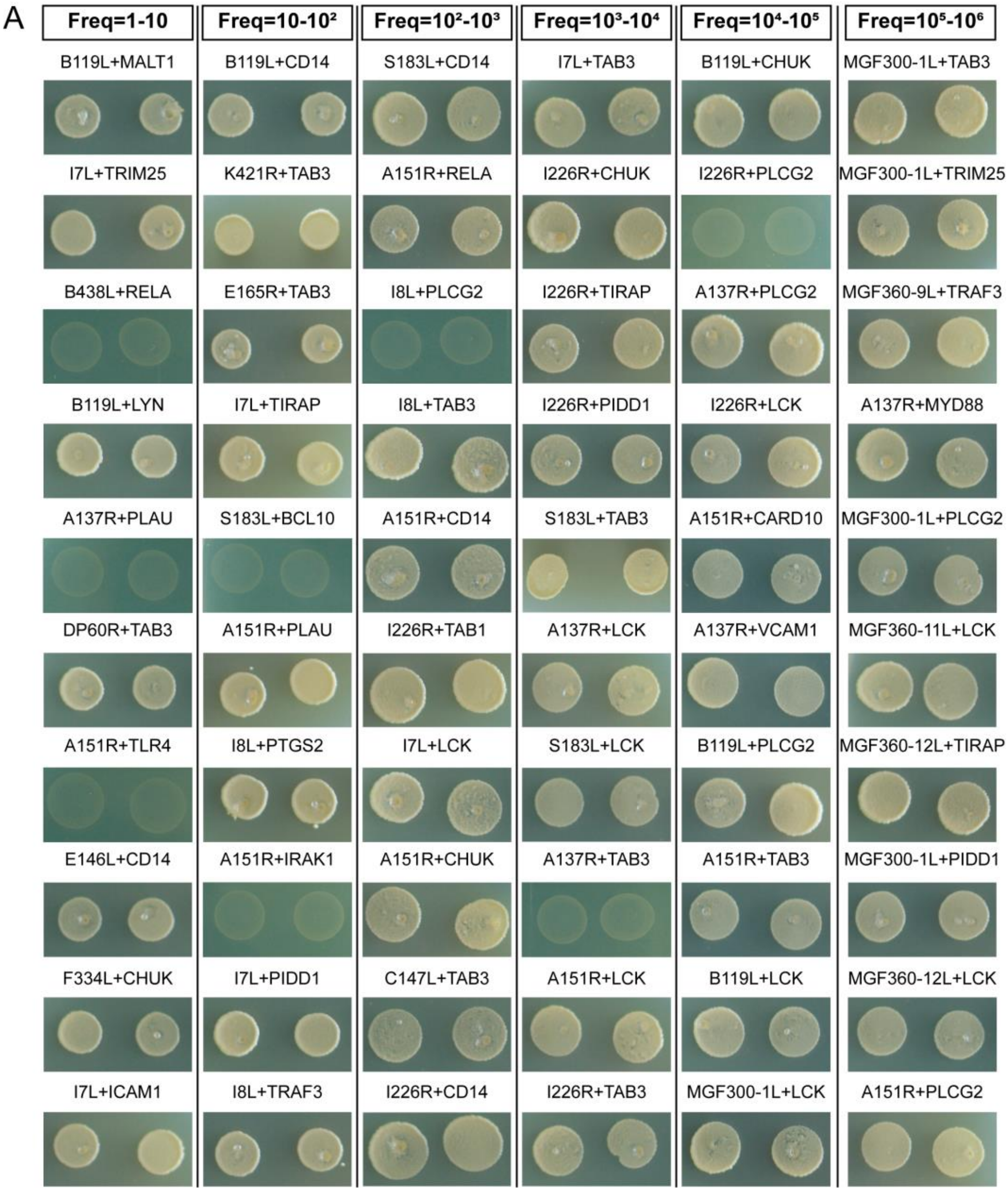

Fig S3. ASFV-host PPI validation by point-to-point Y2H Point-to-point Y2H for randomly selected protein interaction pairs. The illustrated Y2H results were partial results from 548 re-tested PPI listed in Table S4. Freq, frequency.

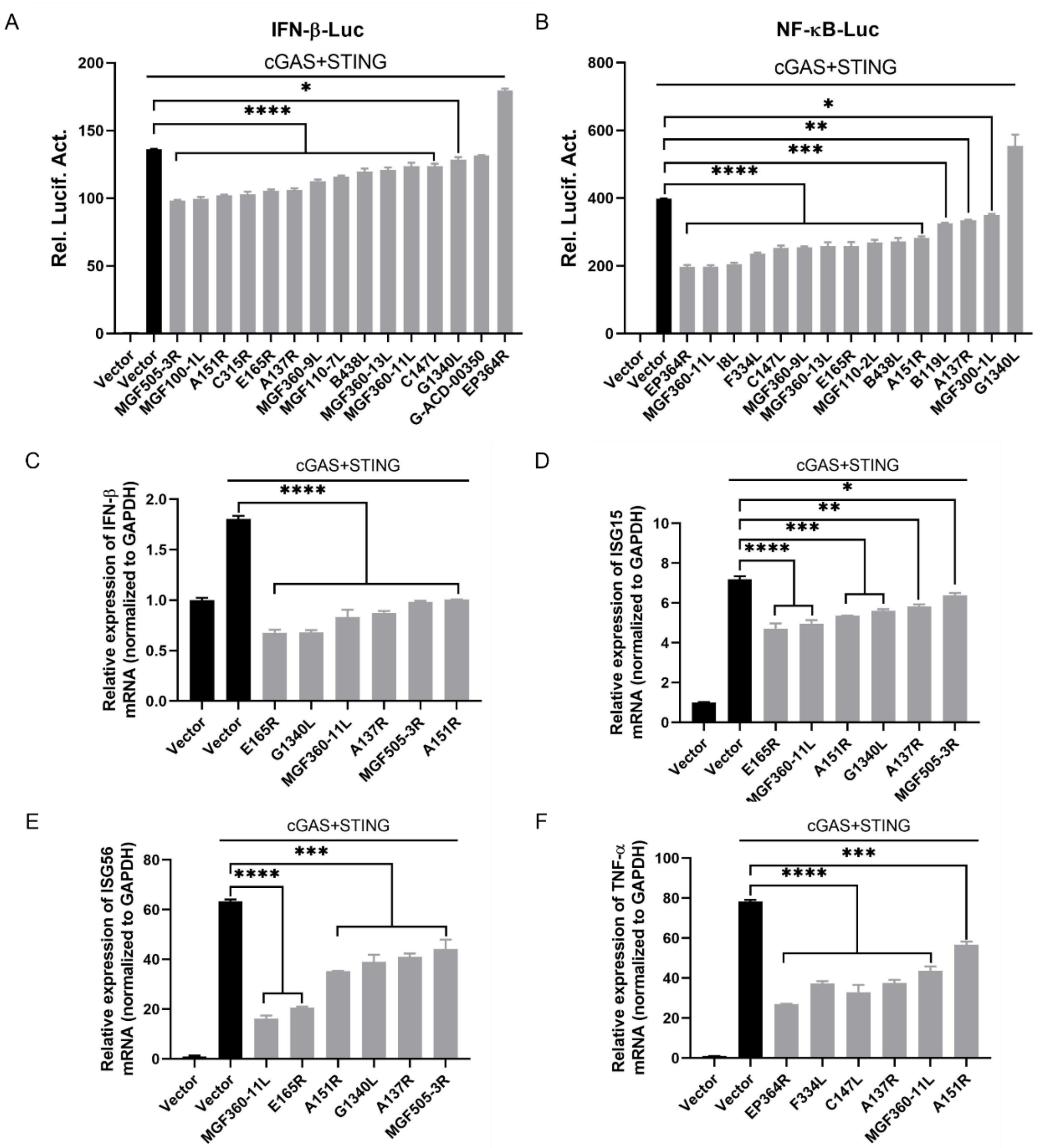

Fig S4. The effect of ASFV proteins on cGAS-STING pathway. (A-B) HEK293T cells were transfected with IFN-β-Luc (A) or NF-κB-Luc (B) reporter, pRL-TK reporter, HA-cGAS (swine) and HA-STING (swine), together with different plasmid expressing HA-ASFV respectively. At 24 hpt, Luc activities were evaluated through the dual-luciferase assay. (C-E) HEK293T cells were transfected with HA-cGAS (swine) and HA-STING (swine), together with ASFV gene eukaryotic expression plasmid (HA-A151R, MGF360-11L, G1340L, A137R, E165R, or MGF505-3R) for 24 h. The mRNA levels of IFN-β (C), ISG15 (D) and ISG56 (E) in the HEK293T cells were analyzed by qRT-PCR assay. (F) HEK293T cells were transfected with HA-cGAS (swine) and HA-

STING (swine), together with ASFV gene eukaryotic expression plasmid (HA-MGF360-11L, A151R, EP364R, C147L, A137R or F334L) for 24 h. The mRNA levels of TNF-α in the HEK293T cells were analyzed by qRT-PCR assay. ****, $p < 0.0001$; ***, $p < 0.001$; **, $p < 0.01$; *, $p < 0.05$ (one-way ANOVA). Data are represented as mean ± SEM.

Table S1. Primers and gene sequences for the construction of host prey library, ASFV bait library, and ASFV prey library.

Table S2. Detailed ASFV-host PPIs between 77 ASFV proteins and 590 host immune pathway proteins.

Table S3. Detailed ASFV-host PPIs for 28 sub-networks.

Table S4. Detailed ASFV-ASFV PPIs between 98 ASFV proteins.

Table S5. Detailed PPIs between ASFV virulent proteins and host.

Table S6. Detailed interaction information between MGF505-3R, MGF360-12L, and IL-1B.

Table S7. Detailed ASFV-ASFV PPIs for early and late ASFV proteins.

Table S8. Protein sequence alignment results of 590 host proteins in the ASFV-host PPI network between domestic pigs (*Sus scrofa*, *S. scrofa*) and warthogs (*Phacochoerus Africanus*, *P. africanus*).

Table S9. Primers for ASFV eukaryotic expression plasmids construction and for qRT-PCR.
